# Differential Effect of Acute and Chronic Exercise on Cardiac Angiogenesis Regulator: The Role of mRNA HIF-1*α* and Its Negative Regulators of In Vivo Study

**DOI:** 10.1155/crp/6348392

**Published:** 2025-11-27

**Authors:** Putri Karisa, Nova Sylviana, Hanna Goenawan, Hanny Primadini Fitrianti, Setiawan

**Affiliations:** ^1^Doctoral Program in Medical Science, PMDSU Batch VIII, Faculty of Medicine, Universitas Padjadjaran, Bandung, Indonesia; ^2^Department of Biomedical Sciences, Faculty of Medicine, Universitas Padjadjaran, Bandung, Indonesia; ^3^Physiology Molecular Laboratory, Biology Activity Division, Central Laboratory, Universitas Padjadjaran, Sumedang, Indonesia; ^4^Center of Sport Science, Wellness, and Longevity, Graduate School, Universitas Padjadjaran, Bandung, Indonesia; ^5^Biomedical Science Master Program, Faculty of Medicine, Universitas Padjadjaran, Bandung, Indonesia

**Keywords:** angiogenesis, cardiovascular health, exercise, factor-inhibiting HIF-1, HIF-1*α*, prolyl hydroxylase domain

## Abstract

**Introduction:**

Angiogenesis is a critical adaptation to regular physical exercise, primarily driven by hypoxia-inducible factor-1 alpha (HIF-1*α*). However, prolonged exercise has been associated with the downregulation of HIF-1*α*, potentially mediated by increased expression of its negative regulators, prolyl hydroxylase domain (PHD) and factor-inhibiting HIF-1 (FIH).

**Objectives:**

This study aimed to investigate the effects of short-term (acute) and long-term (chronic) moderate-intensity exercise on HIF-1*α*, PHD, and FIH mRNA expression in Wistar rat hearts.

**Methods:**

Twenty Wistar rats (age: 8 weeks, body weight: 200–250 g) were divided into four groups: acute control (AC) (15 days) (*n* = 5), acute exercise (AE) (15 days) (*n* = 5), chronic control (CC) (8 weeks) (*n* = 5), and chronic exercise (CE) (8 weeks) (*n* = 5). The exercise groups underwent moderate-intensity treadmill exercise with 20 m/min for 30 min each day for 5 times a week. At the end of the experiment, rats were sacrificed 1 h (acute group) and 24 h (chronic group) after exercise using isoflurane anesthesia, followed by cervical dislocation. Left ventricular heart muscle samples were collected for mRNA expression analysis of HIF-1*α*, PHD, and FIH using real-time PCR.

**Results:**

Exercise significantly altered the expression of HIF-1*α*, PHD, and FIH. HIF-1*α* mRNA was significantly higher in the AE group versus AC (AC vs AE, *p*=0.006) and in the CE group versus CC (CC vs CE, *p*=0.004). PHD expression likewise increased with exercise (AE vs AC, *p*=0.001; CE vs CC, *p* ≤ 0.001). In contrast, FIH showed no significant differences (acute *p*=0.472; chronic *p*=0.095). Exploratory one-way analyses confirmed overall group effects for HIF-1*α* (*p* ≤ 0.001) and PHD (*p*=0.016), but not for FIH (*p*=0.105).

**Conclusion:**

Chronic moderate-intensity exercise upregulates the expression of HIF-1*α* negative regulators (PHD and FIH) in the myocardium, suggesting a shift from acute hypoxia-driven responses to oxygen-dependent regulation. These findings offer insight into the molecular adaptations of cardiac tissue to prolonged exercise and their potential role in angiogenesis regulation.

## 1. Introduction

Physical activity has many positive benefits for physical health, especially the heart and gut microbiome, which have attracted the attention of many researchers [1]. A sedentary lifestyle has long been recognized as a significant risk factor for cardiovascular disease [2]. Regular physical exercise has been widely acknowledged as an effective non-pharmacological intervention to reduce cardiovascular risk [3]. Engaging in regular physical activity enhances cardiorespiratory fitness, lower hospitalization rates, and improves the overall quality of life for individuals with cardiovascular conditions [4]. Additionally, long-term exercise promotes physiological cardiac hypertrophy, which strengthens the heart muscle by enhancing antioxidant capacity, adapting mitochondrial metabolism, cardiomyocyte signaling, molecular pathways, and structural and functional remodeling of blood vessels [5].

In terms of vascular remodeling, exercise stimulates both angiogenesis (the formation of new blood vessels) and arteriogenesis (the widening of existing vessels). It also influences blood flow, myocardial contraction, and oxygen regulation [6]. Oxygen regulates vascular remodeling by activating growth factors that drive endothelial proliferation, migration, and vascular channel formation, especially under hypoxic conditions [7]. Consequently, hypoxia induced by exercise has been identified as a trigger for angiogenesis [8].

Hypoxia-inducible factor 1-alpha (HIF-1*α*) is one of the factors that play an important role in angiogenesis [9]. Hypoxic conditions have been shown to be a strong stimulus to activate the HIF-1*α* protein that results in capillary growth in cardiac tissue through activation of the vascular endothelial growth factor (VEGF) pathway gene, as occurs during physical exercise [7, 10]. Cardiac adaptations to exercise vary based on the type, intensity, and duration [11]. However, the duration of physical exercise has the strongest influence on outcomes [12, 13].

Previous studies have reported inconsistent effects of exercise on HIF-1*α* expression depending on the duration, intensity, and animal model used. For instance, a 12 week high-intensity protocol increased both HIF-1*α* and VEGF protein expression [6], whereas a 4-week moderate exercise regimen resulted in lower HIF-1*α* expression compared to controls [14]. Similarly, Bellafiore et al. observed that HIF-1*α* peaked at day 15 under low-intensity conditions before declining with prolonged training [7]. These variations may arise from differences in tissue type, species, or whether mRNA or protein expression was assessed. Despite these discrepancies, the collective evidence suggests that HIF-1*α* is dynamically regulated by exercise and may contribute to angiogenic adaptation in the myocardium.

The decreased expression of HIF-1*α* during acute adaptation is likely due to the increased activity of negative regulators of the HIF system. Long-term exercise training is believed to suppress HIF-1*α* through the activation of its negative regulators, such as prolyl hydroxylase domain (PHD) and factor-inhibiting HIF-1 (FIH) [13]. The Lindholm et al. study showed that low HIF-1*α* expression has results that are in line with increased expression of FIH and PHD [15]. Meanwhile, moderate-intensity physical exercise for 8 weeks showed an increase in HIF-1*α* [16]. Although there have been many studies that prove the effect of physical exercise on the expression of HIF-1*α* as a regulator of angiogenesis, most studies only compare differences in intensity that focus on assessing HIF-1*α* expression. Meanwhile, a comprehensive analysis comparing acute and chronic responses in the regulation of HIF-1*α* and its inhibitory factors has never been done, especially in the heart. Based on this, we hypothesized that when HIF-1*α* is expressed as a form of acute adaptation, the expression of FIH and PHD will be suppressed. Vice versa, FIH and PHD expression will increase in the chronic period. This study aimed to investigate the effects of short-term (acute) and long-term (chronic) moderate-intensity exercise on HIF-1*α*, PHD, and FIH mRNA expression in Wistar rat hearts.

## 2. Methods

### 2.1. Animal Model and Experimental Design

Twenty 8-week-old healthy male Wistar rats weighing approximately 200–250 g from the PT Bio Farma Laboratory (Parongpong, West Java, Indonesia) were used in this study. The rats were introduced to the treadmill every day during acclimation. Following a one-week acclimation period, the rats were randomly divided into four groups: acute control (AC) (15 days) (*n* = 5), acute exercise (AE) (15 days) (*n* = 5), chronic control (CC) (8 weeks) (*n* = 5), and chronic exercise (CE) (8 weeks) (*n* = 5). The age of the animals at the end of the study was 10 weeks for the acute group and 16 weeks for the chronic group. To account for the difference in age at the end of the intervention, each exercise group was matched with an age-appropriate control group (AC for AE; CC for CE). Gene expression analysis was therefore performed within each time-stratified pair to ensure that the results reflect the effect of exercise duration rather than age-related differences.

The sample inclusion criteria in this study were healthy rats that completed the exercise according to the protocol. Exclusion criteria include rats that are sick, dead, and refuse to do the exercise. In this study, all rats were able to complete the exercise protocol. The rats were randomly housed under controlled conditions, maintaining an ambient temperature of approximately ±25°C, 12 h light/dark cycle (300 1x light), stable humidity, the environment free from noise and ultrasonic disturbances, and a relative humidity of 40%–70%. Standard diet (Prospets laboratory feed normal diet; 30,294 kcal/100 g) and water were provided ad libitum to all groups. The allocation and implementation of the experiment were conducted blindly by a laboratory assistant. All animal protocols were approved by the Research Ethics Committee of Universitas Padjadjaran (No.315/UN6.KEP/EC/2024) and were performed following the guidelines for the use of laboratory animals in accordance with ARRIVE guidelines (Supporting file 1) [17].

### 2.2. Exercise Protocol

To familiarize the rats with the treadmill, a one-week acclimation process was implemented. On the first day, they were placed on a stationary treadmill for 30 min, and in the subsequent days, they were exposed to a low-speed setting of 8 m/min to help them adjust to the treadmill's sound when it is turned on. There is no increase in speed during the acclimation period. In this study, “acute” and “chronic” exercise protocols were defined based on training duration, following the approach by Bellafiore et al., who demonstrated that HIF-1*α* expression peaked after 15 days of treadmill training and declined with prolonged exercise. Accordingly, the acute group underwent a 15 day resistance training protocol to represent early-phase adaptation, while the chronic group completed 8 weeks of training to represent long-term adaptation.

For moderate-intensity training, rats were placed on the treadmill with the same speed and duration for each treatment group. The designated training speed was 20 m/min for 30 min each day for 5 times a week. The AE group followed this regimen for 15 days and the CE group continued for 8 weeks. The exercise intensity was determined based on lactate accumulation levels and prior research findings [18–20].

### 2.3. Tissue Collection and RNA Extraction

At the end of each protocol, animals were euthanized at different time points: the acute group 1** **h after the final exercise session and the chronic group 24 h after the final session, to distinguish short-term molecular responses from sustained adaptations. Termination was carried out by using isoflurane anesthesia, followed by cervical dislocation. Left ventricular heart muscle samples were collected for mRNA expression analysis of HIF-1*α*, PHD, and FIH using real-time polymerase chain reaction (RT-PCR). Total RNA was extracted using GENEzol reagent, as described by the manufacturer (Geneaid, Taipei, Taiwan). Complementary DNA (cDNA) of HIF-1*α*, PHD, and FIH was synthesized from total RNA using specific reverse primers by reverse transcriptase enzyme, as shown in Table 1.

### 2.4. RT-PCR Analysis

Gene expression levels of HIF-1*α*, PHD, and FIH were analyzed through RT-PCR, with glyceraldehyde-3-phosphate dehydrogenase (GAPDH) serving as a housekeeping gene for normalization (Supporting 2). PCR was performed by mixing 5 µL 2x Sensifast SYBR mix, 0.4 µL primer forward, 0.4 µL primer reserve, 0.2 µL RNase inhibitor, 0.1 µL reverse transcriptase enzyme, 2 µL RNA template, and 1.9 µL DEPC for a total volume of 10 µL (SensiFAST SYBR Lo-ROX One-Step Kit Bioline, USA). RT-PCR was performed using AriaMx SYBR RT-PCR technology (Agilent Technologies, USA). The primer sequences were rats-based and designed using the Primer-BLAST tool (NCBI) (Integrated DNA Technologies, Coralville, Iowa, USA). The relative expression was determined using the 2^−ΔΔCt^ method as the reference.

### 2.5. Statistical Analysis

Statistical analysis was conducted using GraphPad Prism 9. The Shapiro–Wilk test was used to assess the normal distribution of the data, since the sample size was small. All data are presented as mean ± standard error of the mean (SEM), unless otherwise stated. SEM was used to reflect the precision of the estimated group means. Because the acute (15 day) and chronic (8 week) protocols involved different animal cohorts, comparisons were performed within each time stratum (AC vs AE and CC vs CE) rather than across all four groups. Normally distributed data were analyzed using independent *t*-tests, while non-normal data used the Mann–Whitney *U* test. An exploratory one-way ANOVA test (for normally distributed data) and the Kruskal–Wallis test (for non-normally distributed data) were also performed to illustrate overall group trends. A *p* value < 0.05 was considered statistically significant.

## 3. Results

### 3.1. Body Weight (BW) and Heart Weight (HW)

**Figure 1:**
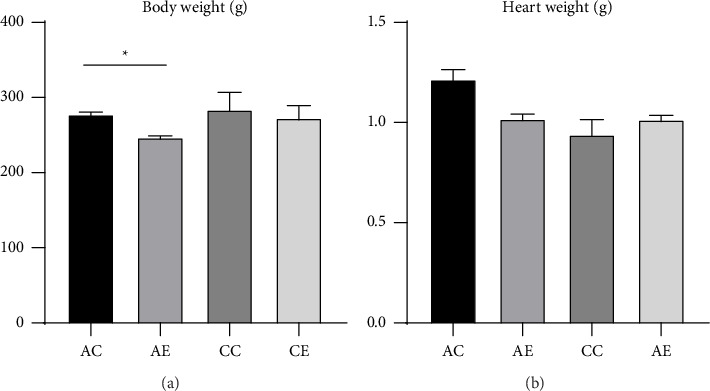
Body and heart weights. (a) Body weights of rats in (AC, AE, CC, CE groups). (b) Heart weights of rats. Notes: AC, acute control; AE, acute exercise; CC, chronic control; CE, chronic exercise. Values are presented as mean ± SEM.

Table 2 presents the overall BW and HW measurements among the groups. The analysis showed a significant increase in BW across all groups after the intervention period (*p*=0.001), indicating normal physiological growth during the study. Although the mean HW appeared slightly higher in the AC group (1.2 ± 0.06 g) compared to the other groups, this difference was not statistically significant (*p*=0.094). Similarly, the HW-to-BW (HW/BW) ratio, which serves as an indicator of relative cardiac hypertrophy, did not differ significantly among groups (*p*=0.354). These results suggest that both acute and CE interventions did not induce a substantial change in relative cardiac mass, and the observed increase in BW likely reflects general somatic growth rather than exercise-induced hypertrophy (Figure 1).

### 3.2. HIF-1*α*, FIH, and PHD mRNA Expression in AE vs CE

**Figure 2:**
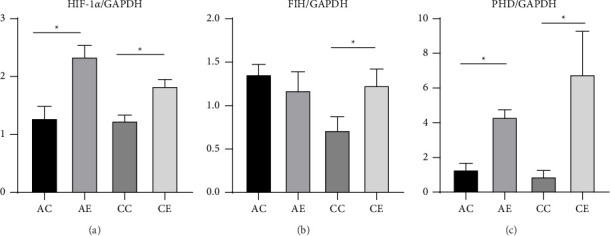
HIF-1*α*, FIH, and FHD mRNA expressions in heart. Notes: (a) HIF-1*α* group mRNA level was quantified by RT PCR. (b) FIH group mRNA level was quantified by RT PCR. (c) PHD group mRNA level was quantified by RT PCR. Data are expressed as mean ± SEM. ^∗^*p* < 0.05 compared to control group (one-way ANOVA with post hoc Tukey test).

The Shapiro–Wilk test showed that HIF-1*α* and PHD data were normally distributed, whereas FIH data were nonparametric. Therefore, comparisons were conducted within each time stratum between age-matched control and exercise groups. HIF-1*α* expression was significantly higher in the AE group compared with its control (AC vs AE, *p*=0.006) and in the CE group compared with its control (CC vs CE, *p*=0.004), with an exploratory one-way ANOVA confirming overall group differences (*p* ≤ 0.001). FIH expression did not differ significantly between either acute or chronic comparisons (Mann–Whitney, *p*=0.472 and 0.095, respectively), and the Kruskal–Wallis test indicated no global difference (*p*=0.105). In contrast, PHD mRNA levels were markedly elevated after exercise, both in AE vs AC (*p*=0.001) and CE vs CC (*p* ≤ 0.001), supported by ANOVA significance (*p*=0.016). Collectively, moderate-intensity acute and CE upregulated HIF-1*α* and its regulator PHD, while FIH remained unchanged, suggesting an adaptive modulation of myocardial oxygen-sensing pathways. The detailed data can be found in Table 3 and Figure 2.

## 4. Discussion

HIF-1*α* is a transcription factor that responds to oxygen fluctuations by regulating genes involved in angiogenesis and metabolism [21]. During acute hypoxia, limited oxygen or increased mitochondrial reactive oxygen species (ROS) inhibits HIF-1*α* hydroxylation, stabilizing the protein and activating targets such as VEGF [4, 22–24]. Exercise serves as a potent physiological trigger of local hypoxia, and previous studies have shown that AE activates the HIF-1 pathway, upregulating VEGF and erythropoietin (EPO) while decreasing von Hippel–Lindau (VHL) protein levels to promote capillary formation [25]. Exercise acts as a potent physiological trigger of transient hypoxia, activating the HIF-1 pathway and promoting capillary formation through the suppression of VHL–mediated degradation. In our study, HIF-1*α* mRNA expression peaked in the 15 day (acute) exercise group, indicating a rapid hypoxic response within cardiac tissue. This transient oxygen deficit stabilizes HIF-1*α* by suppressing oxygen-dependent post-translational modifications, enabling the transcription of genes that enhance oxygen delivery and metabolic adaptation [26]. However, in the chronic (8 week) exercise group, HIF-1*α* mRNA expression decreased relative to the acute phase but remained higher than in sedentary controls (*p*=0.002), suggesting a shift from acute hypoxia-induced signaling to long-term adaptation. Previous studies have shown that repeated exercise improves oxygen utilization and vascular efficiency, attenuating hypoxia sensitivity and reducing HIF-1*α* induction over time [12, 13, 27–30]. This adaptive downregulation is supported by increased mitochondrial density and oxidative metabolism, which minimize the hypoxic drive for HIF-1*α* activation.

The negative regulators of HIF-1*α*, FIH, and PHD enzymes play essential roles in maintaining oxygen homeostasis. FIH hydroxylates HIF-1*α* at Asn803, blocking p300/CBP coactivator binding and inhibiting transcriptional activation [31–33]. In this study, FIH expression did not significantly differ between AE and control groups, although it tended to decrease as HIF-1*α* increased, implying suppressed FIH activity during early hypoxia. This is consistent with Zhang et al. [34], who reported that loss of FIH activity facilitates full HIF-1*α* transcriptional activation under low-oxygen conditions. Conversely, after 8 weeks of exercise, FIH mRNA expression increased significantly (*p*=0.047), likely reflecting enhanced oxygenation following capillary growth and improved perfusion. This elevated oxygen availability reactivates FIH-mediated hydroxylation, limiting HIF-1*α* activity and stabilizing oxygen-dependent homeostasis [35–38].

Under normal oxygen conditions (normoxia), HIF-1*α* subunits undergo hydroxylation at proline residues (Pro402 and Pro564) by PHD enzymes, which promote ubiquitination by VHL ligase, leading to proteasomal degradation. Additionally, HIF-1*α* transactivation is inhibited in an oxygen-dependent manner by FIH-mediated asparaginyl hydroxylation [39, 40]. This oxygen-dependent hydroxylation plays a critical role in regulating HIF-1*α* stability and transcriptional activity [39]. PHD enzymes belong to the family of 2-oxoglutarate (*α*KG)-dependent, nonheme iron-binding dioxygenases and require oxygen, 2-oxoglutarate, iron (Fe), and ascorbate as cofactors. While oxygen is the primary regulator of HIF-1*α* hydroxylation, other factors such as Fe, ascorbate, and 2-OG also contribute significantly to its degradation [41, 42]. PHD activity regulates HIF-1*α* stability in response to oxygen availability, and under hypoxic conditions, PHD inhibition allows HIF-1*α* stabilization, facilitating cellular adaptation [12]. This aligns with in vitro findings by Epstein et al., who demonstrated that PHD activity decreases when oxygen levels drop from 21% to 0%, accompanied by a reduction in oxygen partial pressure [43].

Interestingly, PHD mRNA expression also increased significantly in the AE group, which appears paradoxical given its expected suppression under hypoxia [39, 43, 44]. Similar findings were reported by Stiehl et al. [45] and D'Angelo et al. [46], showing transient PHD upregulation even in hypoxic conditions, suggesting that PHDs act as part of a feedback mechanism to terminate the hypoxic response once partial reoxygenation occurs. The upregulation observed in our study may therefore represent an adaptive counter-regulatory response, aiming to fine-tune HIF-1*α* stability during fluctuating oxygen tensions caused by short-term exercise. Collectively, these findings provide novel evidence that acute resistance exercise induces transient cardiac hypoxia and activates the HIF-1*α* pathway, while CE leads to a compensatory increase in its negative regulators, FIH and PHD, reflecting vascular and metabolic adaptation. This highlights a dynamic balance between oxygen-sensing and oxygen-restoring mechanisms within the heart, an adaptive continuum rarely characterized at the cardiac level in the context of resistance exercise.

Although direct measurements of VEGF and capillary density were not performed in this study, previous research has consistently shown that activation of HIF-1*α* by exercise leads to increased VEGF expression and subsequent angiogenesis in cardiac and skeletal muscle. Stabilized HIF-1*α* dimerizes with HIF-1β and binds hypoxia response elements (HREs) in target gene promoters, including the VEGF promoter, to drive transcriptional upregulation of proangiogenic factors [47, 48]. During intense hypoxia, inhibition of PHD activity has been shown to stabilize HIF-1*α* and enable VEGF transcription. Additionally, the role of hydroxylated FIH can prevent the recruitment of the CBP/p300 transcription coactivator, thereby reducing HIF-dependent transactivation without altering the stability of the HIF-*α* protein [33]. Manipulation of PHD or FIH activity (pharmacological inhibitors, loss of generic function) can alter HIF target genes, including VEGF. The study by Cirillo et al. revealed that increased PHD levels can inhibit HIF-1-mediated VEGF transcription, while PHD inhibition rescues VEGF expression in skeletal muscle [49]. Furthermore, higher plasma VEGF concentrations due to PHD inhibition in mouse models showed lower infarct sizes [50]. Research on rats with HIF-1 deficiency mediated by PHD and FIH was unable to survive during the embryonic phase due to a significant decrease in VEGF, which is important in angiogenesis and vasculogenesis [51]. VEGF is a target of HIF-1*α* whose expression can be inhibited by FIH activation [52]. HIF-1*α* induces VEGF and angiopoietin-2 (Ang-2) to regulate angiogenesis by directing adult endothelial cell migration to hypoxic zones [53]. Thus, inhibition of HIF-1 by FIH not only inhibits VEGF but also Ang-2, which plays a role in increasing endothelial permeability to allow VEGF-induced cell migration [54, 55]. Therefore, these findings provide causal evidence that this hydroxylase is the main inhibitor of exercise- or hypoxia-induced angiogenesis.

**Figure 3:**
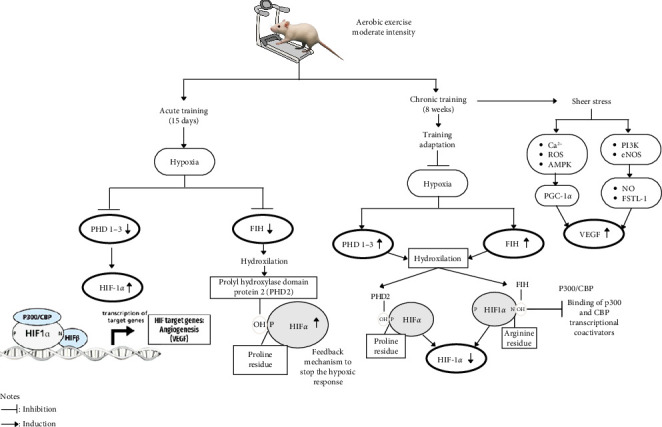
Proposed mechanism describing the role of angiogenesis regulators in acute and chronic adaptation to physical training.

In the 8-week exercise group (chronic adaptation), a significant increase in cardiac PHD levels was observed in the intervention group compared to the control, aligning with its normoxic expression. Under sufficient oxygen conditions, PHD hydroxylates the prolyl residues of HIF-1*α*, leading to its degradation. This oxygen availability may result from improved oxygenation due to an expanded capillary network formed in response to hypoxia in ischemic muscle. Low oxygen tension (pO_2_) triggers angiogenesis by upregulating VEGF, activated through hypoxia-response elements regulated by HIF-1*α* [44]. This is shown by a decrease in HIF-1*α* levels along with an increase in PHD levels in the moderate-intensity physical exercise group for 8 weeks which is a form of adaptation in blood vessels. This is similar to conducting a cross-sectional study comparing elite athlete groups and moderately active individuals. Consequently, PHD and FIH expression levels have been found to be higher in elite athletes than in moderately active individuals, as observed in skeletal muscle biopsies [15]. To understand how the mechanisms of acute and chronic adaptation in modulating angiogenesis regulators and inhibiting factors after moderate-intensity exercise can be seen in Figure 3 (Figure 3).

It is important to note that PHD and FIH regulate HIF-1*α* primarily through post-translational hydroxylation rather than transcriptional control. Therefore, the observed mRNA expression patterns in this study likely reflect early transcriptional adaptations rather than direct regulation of HIF-1*α* protein stability. Although mRNA and protein levels do not always correlate, transcriptional changes in these regulatory enzymes may indicate cellular readiness to modulate HIF-1*α* activity in response to exercise-induced hypoxia. Future studies are warranted to validate these findings at the protein level using Western blotting or immunohistochemistry.

The absence of significant differences in heart weight and HW/BW ratio across groups indicates that the exercise duration or intensity was insufficient to elicit overt cardiac hypertrophy. Such findings are consistent with evidence that early or moderate training primarily induces functional and molecular remodelling, such as enhanced perfusion and metabolic efficiency, rather than structural enlargement [56, 57]. Despite the lack of gross hypertrophy, molecular data revealed upregulated HIF-1*α* and concurrent downregulation of its negative regulators, PHD and FIH, suggesting a shift toward a hypoxia-responsive cellular state [58, 59]. These findings highlight that the heart's early adaptation to resistance exercise involves molecular activation of oxygen-sensing and angiogenic pathways, representing a preparatory phase for later morphological remodeling with continued training stimulus.

## 5. Conclusion

This study demonstrates that acute and CE elicit distinct regulatory effects on cardiac angiogenesis in Wistar rats, primarily through HIF-1*α* signaling and its negative regulators. AE promotes HIF-1*α* activation, while CE enhances adaptive control by upregulating PHD and FIH, suggesting potential involvement in maintaining vascular balance during prolonged training. These findings highlight a possible compensatory mechanism through HIF-1*α* inhibition, although direct vascular functional assessments are required to confirm this effect. This study is limited by the lack of protein-level analyses of key angiogenic markers, such as VEGF and PGC-1*α*, which could strengthen the mechanistic interpretation. Additionally, there was an age difference between the acute (10 weeks) and chronic (16 weeks) groups at the time of tissue collection, which may influence gene expression independently of exercise. However, age-matched control groups were provided for each intervention group (acute and chronic) to allow stratified comparisons. Despite this, caution is warranted when interpreting differences between the acute and chronic groups, as age-related factors may still contribute to the observed results. Future studies are needed to comprehensively assess angiogenesis mechanisms using high-accuracy protein-level methods such as Western blotting and to determine the long-term effects of exercise-induced hypoxic regulation in the heart, including angiogenesis markers.

## Figures and Tables

**Table 1:** Primer sequences.

Gene	Primer	Sequences
HIF-1*α*	Forward	5′-CTCCCATACAAGGCAGCAGAAA-3′
	Reverse	5′-CAAAACAACCAACAGAAACGAAAC-3′

FIH	Forward	5′-GTGCCAGCACCCATAAGTT-3′
	Reverse	5′-CGCGCTGCTGTATAGCTT-3′

PHD	Forward	5′-GGCCGCTGTATCACCTGTA T-3′
	Reverse	5′-TTCTGCCCTTTCTTCAGCAT-3′

GAPDH	Forward	5′-GTTACCAGGGCTGCCTTCTC-3′
	Reverse	5′-GATGGTGATGGGTTTCCCGT-3′

*Note:* mRNA, messenger ribonucleic acid.

Abbreviations: FIH, factor-inhibiting HIF-1; GAPDH, glyceraldehyde-3-phosphate dehydrogenase; HIF-1*α*, hypoxia-inducible factor-1 alpha; PHD, prolyl hydroxylase domain.

**Table 2:** Body and heart weight.

Groups	Body weight (g)				Heart weight		HW/BW ratio	
	BW before (*g*)	BW after (*g*)	ΔBW (*g*)	*p* value	HW (*g*)	*p* value	HW/BW ratio	*p* value
AC	242.2 ± 8.8	277.2 ± 3.4	35	0.001^∗^	1.2 ± 0.06	0.094	0.0043	0.354
AE	214.8 ± 4.7	246.8 ± 2.3	32		1.0 ± 0.04		0.0042	
CC	229.6 ± 3.9	283.6 ± 2.3	54		0.92 ± 0.1		0.0034	
CE	221.4 ± 5.8	272.4 ± 1.7	51		1.00 ± 0.03		0.0039	

Abbreviations: BW, body weight; HW, heart weight.

^∗^Statistically significant difference compared with the control group (*p* < 0.05).

**Table 3:** HIF-1*α*, FIH, and PHD mRNA expression in heart.

Groups	HIF-1*α*^∗^				FIH^†^			PHD^∗^	
	Mean ± SEM	Independent *t*-test	ANOVA test	Mean ± SEM	Mann–Whitney	Kruskal–Wallis test	Mean ± SEM	Independent *t*-test	ANOVA test
AC	1.280 ± 0.205	0.006^‡^	< 0.001^‡^	1.360 ± 0.114	0.472	0.105	1.304 ± 0.359	0.001^‡^	0.016^‡^
AE	2.344 ± 0.194			1.176 ± 0.215			4.328 ± 0.425		
CC	1.238 ± 0.097	0.004^‡^		0.716 ± 0.154	0.095		0.898 ± 0.355	< 0.001^‡^	
CE	1.834 ± 0.114			1.232 ± 0.188			6.788 ± 2.480		

*Note:* Data are presented as mean ± SEM. Normality was assessed using the Shapiro–Wilk test. Comparisons were performed within each time stratum (AC vs AE; CC vs CE) using independent *t*-tests for normally distributed data or Mann–Whitney *U* tests for non-normal data. Exploratory one-way ANOVA (or Kruskal–Wallis for nonparametric data) was also performed to illustrate global trends. *p* < 0.05 was considered statistically significant.

Abbreviations: AC, acute control; AE, acute exercise; CC, chronic control; CE, chronic exercise.

^∗^Normal distribution (*p* > 0.05), continued with a significance test using an ANOVA test and Independent *t*-test.

^†^Distribution is not normal (*p* < 0.05), continued with a significance test using the Kruskal–Wallis test and Mann–Whitney test.

^‡^Significant data (*p* value < 0.05).

## Data Availability

The data that support the findings of this study are available from the corresponding author upon reasonable request.

## Supporting Information

Supporting Information 1 Supporting Information 1. Supporting Table 1: ARRIVE Guidelines Checklist–A completed checklist outlining compliance with the Animal Research: Reporting of In Vivo Experiments (ARRIVE) 2.0 guidelines, submitted to ensure transparency and reproducibility in animal research reporting.

Supporting Information 2 Supporting Information 2. Supporting Table 2: The mRNA Expression of GAPDH as Housekeeping Gene.
