# Supplementary figures and images for: Differential Effect of Acute and Chronic Exercise on Cardiac Angiogenesis Regulator: The Role of mRNA HIF-1α and Its Negative Regulators of In Vivo Study
Source: Cardiol Res Pract. 2025 Nov 27;2025:6348392. doi: 10.1155/crp/6348392 (PMC12677996; doi:10.1155/crp/6348392)

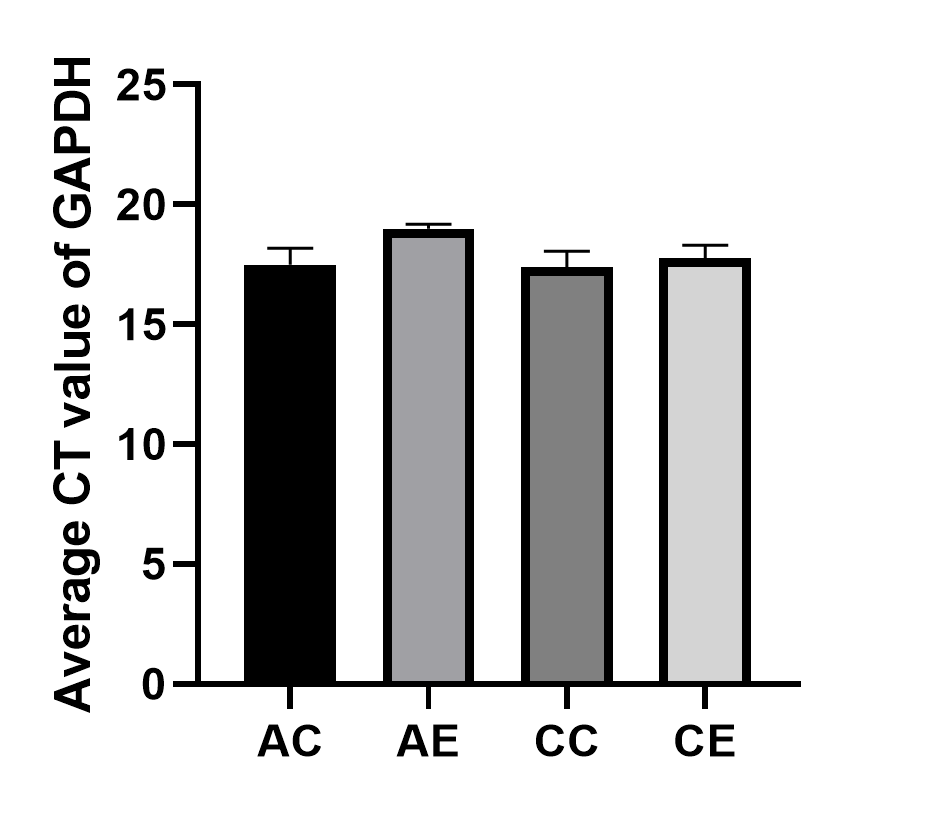

Supplement: Supporting Information 2 — Supporting Table 2: The mRNA Expression of GAPDH as Housekeeping Gene. [file 6348392.f2.docx]
